# Alarm Tones, Voice Warnings, and Musical Treatments: A Systematic Review of Auditory Countermeasures for Sleep Inertia in Abrupt and Casual Awakenings

**DOI:** 10.3390/clockssleep2040031

**Published:** 2020-10-20

**Authors:** Stuart J. McFarlane, Jair E. Garcia, Darrin S. Verhagen, Adrian G. Dyer

**Affiliations:** 1 Bio-Inspired Digital Sensing Lab (BIDS), School of Media and Communication, Digital Ethnography Research Centre (DERC), RMIT University, Melbourne, VIC 3001, Australia; jair.garcia@rmit.edu.au (J.E.G.); adrian.dyer@rmit.edu.au (A.G.D.); 2 School of Design, RMIT University, Melbourne, VIC 3001, Australia; darrin.verhagen@rmit.edu.au

**Keywords:** sleep inertia, sleep, auditory countermeasures, human performance, emergency awakenings, non-emergency awakenings, alarm tones, voice signals, music

## Abstract

Sleep inertia is a measurable decline in cognition some people experience upon and following awakening. However, a systematic review of the current up to date evidence of audio as a countermeasure has yet to be reported. Thus, to amend this gap in knowledge, the authors conducted this systematic review beginning with searches in three primary databases for studies published between the inception date of each journal and the year 2020. Search terms contained “Sleep Inertia” paired with: “Sound”; “Noise”; “Music”; “Alarm”; “Alarm Tone”; “Alarm Sound”; “Alarm Noise”; “Alarm Music”; “Alarm Clock”; “Fire Alarm”, and “Smoke Alarm”. From 341 study results, twelve were identified for inclusion against a priori conditions. A structured narrative synthesis approach generated three key auditory stimulus themes-(i) Noise, (ii) Emergency tone sequences; Voice Alarms and Hybrids, and (iii) Music. Across themes, participants have been assessed in two situational categories: emergency, and non-emergency awakenings. The results indicate that for children awakening in emergency conditions, a low pitch alarm or voice warnings appear to be more effective in counteracting the effects of sleep inertia than alarms with higher frequencies. For adults abruptly awakened, there is insufficient evidence to support firm conclusions regarding alarm types and voice signals. Positive results have been found in non-emergency awakenings for musical treatments in adults who preferred popular music, and alarms with melodic qualities. The results observed reflect the potential for sound, voice, and musical treatments to counteract sleep inertia post-awakening, and emphasize the requirements for further research in this domain.

## 1. Introduction

“I’ve woken up actually holding my pager and actually pressed the button on the pager and went straight back to sleep again”. A firefighter describing an incident when responding to their on-call auditory alarm during an emergency [1].

Sleep Inertia (*SI*) is an experienced and quantifiable reduction in human performance that occurs upon and post-awakening [2,3,4]. The performance decrements observed during *SI* have been shown to expire typically within 30 min; however, durations of up to 4 h have been reported [2,5,6,7,8,9]. Research suggests that the duration and severity may be linked to several factors, including sleep stage at awakening [6,10,11,12,13,14], sleep deprivation [15,16,17,18,19,20], and time of day [14,21,22]; however, these factors do not guarantee that *SI* will be abolished in all circumstances. The cognitive impairments examined to be associated with *SI* include, yet are not limited to, reaction time (RT) [23,24,25], accuracy [19], memory [26] and complex decision making [11,27]. In a real-world context, the reductions in human functioning as a result of *SI* pose an increased risk for general activities occurring post-awakening (e.g., driving, bike riding), and personnel operating in high-risk conditions, including first responders (e.g., police, and the fire brigade), and logistics (air, road, and sea) [1,28,29]. Thus, *SI* is frequently highlighted as a phenomenon requiring improved understanding and further research spanning many fields to mitigate such occurrences [30,31,32,33,34,35,36].

Preemptive measures seek to reduce the envisioned impacts of *SI* through managed sleep epochs and napping [37,38,39,40]. However, these approaches do not compensate for unforeseen events, emergencies, or lifestyle commitments that may inhibit the ability to structure sleep-wake cycles or promote thorough sleep hygiene. For example, during on-call working arrangements where events cannot be predicted, a respondent must take action regardless of the sleep stage prior to awakening, time of day, or prior sleep duration [1]. Furthermore, in regular day to day awakenings, sleep deprivation which is a known factor that enhances *SI* [41,42] is increasingly becoming a prevalent occurrence within populations [43,44]. Thus, post-awakening countermeasures are conceptualized and researched to address these concerns by considering habitual behaviors, environmental conditions, and awakening routines. These include caffeine intake [23,45,46,47,48], lighting treatments [25,47,49,50,51], temperature [52,53], post-awakening routines [47,54], and applied stress [55]. Concerning the potential for sound to counteract *SI*, existing reviews of *SI* literature contain four instances where auditory countermeasures have been acknowledged [2,3,4,56]; however, within these four reviews, only two original research studies are identified for discussion that interrogate noise and music [57,58].

The rationale for auditory signals to have the potential to counteract *SI* stems from research in the fields of auditory perception and cognition. It has been shown that in alert humans noise (e.g., White noise: A random auditory signal having equal intensity at different frequencies) [59,60,61], environmental sounds (e.g., Boiling water) [62], and music (e.g., Rock; Classical) can potentially enhance arousal and improve task performance [63,64,65,66,67]. In the awakening of sleeping humans, auditory arousal thresholds (AATs) have been researched to understand the time it may take an individual to awaken in response to different alarm tone designs and signal loudness (measured in Decibel [dB]) (See Thomas and Bruck [68] for a review). However, the cognitive benefits attributed to the soundings post-awakening are infrequently measured or reported. By undertaking this systematic review, we aim to update and expand the existing empirical-based evidence that reports post-awakening auditory effects on *SI*, and by doing so, inform researchers and industry of auditory approaches that may be further examined or implemented.

## 2. Methods

In the production of this review, the authors (SJM, AGD) applied the PRISMA statement guidelines where applicable [69] (Table S1). Secondly, an a priori search strategy was developed in accordance to the PRISMA-P [70] guidelines (Item No. 10).

### 2.1. Eligibility Criteria 

Eligible articles were required to report original research on the analysis of *SI* in subjects post-awakening to auditory stimuli (objectively or subjectively). All laboratory, field and mixed-method studies employing either electroencephalographic (EEG) or non-EEG confirmed awakenings were included. No limitations were imposed on the types of auditory stimuli tested, nor participant age, sex, or gender. All eligible articles were required to be published in English, report healthy (unimpaired) human experimental results, and be published in peer-reviewed or refereed journals. All publications dates were accepted. Qualitative analysis studies and reviews (not original research) were excluded.

### 2.2. Information Sources, Search Strategy and Study Selection

**Figure 1:**
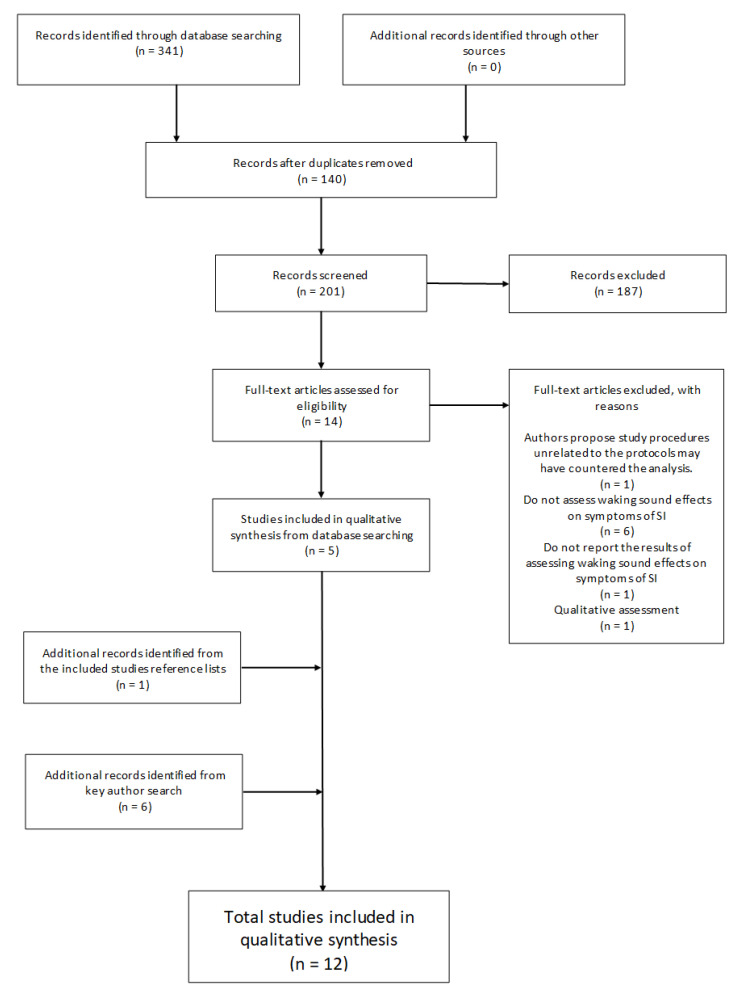
Flowchart illustrating the review selection process.

The primary search strategy employed three electronic databases (ProQuest, PubMed, and Scopus) on the 15 March 2020 to search for the following term “Sleep Inertia” coupled with: “Sound”; “Noise”; “Music”; “Alarm”; “Alarm Tone”; “Alarm Sound”; “Alarm Noise”; “Alarm Music”; “Alarm Clock”; “Fire Alarm”, and “Smoke Alarm”. This resulted in *n* = 341 articles. One hundred and forty (*n* = 140) duplicate articles were identified and removed from the total leaving two hundred and one (*n* = 201) for screening. A further (*n* = 187) records were excluded that did not meet the inclusion criteria based on Title, Abstract and Methodological screening. The remaining fourteen (*n* = 14) articles were reviewed through full-text assessment resulting in nine (*n* = 9) omissions based on the inclusion/exclusion criteria. One article was excluded due to unrelated study procedures that may have confounded results which the authors acknowledge and attribute to the insignificant findings [55]. Each remaining article (*n* = 5) reference list was then manually assessed for additional records which resulted in the identification of *n* = 1 study to be included. Additionally, all lead authors’ (Dorothy Bruck; Mitsuo Hayashi; Stuart J. McFarlane; Gary A. Smith; Patricia Tassi) publication records from the six articles identified for inclusion were individually searched in Google Scholar to verify that no associated literature may have been overlooked through publication and search term bias [71]. Google scholar was chosen as the preferred electronic search engine as it has been shown to be the most comprehensive, yet sometimes imprecise tool currently available [72]. The search was conducted on the 21 May 2020. After excluding duplicates from the search process outlined above, six (*n* = 6) additional articles were identified that adhered to the inclusion/exclusion criteria, extending the total number of articles for review to twelve (*n* = 12). See Figure 1 for the screening flow diagram.

### 2.3. Data Collection Process and Data Items

To extract data from each selected article, the researchers used forms to tabulate specific attributes of all studies which highlight the relevant information necessary to assist in the aims of the review [73]. This information was categorized into the following sections: Author, Number of participants (*n*), Age, Sex or Gender, Setting, Study design, Sleep duration, Awakening time, Stimulus, Decibel (dB), EEG-confirmed awakening (Yes/No), Objective measure, and Subjective measure; where the inserted (Yes/No) indicates a binary categorization whilst other factors were expressed as reported variables. See Table 1 for a summary of the articles selected.

### 2.4. Analysis of Bias

Due to the observed inconsistencies (i.e., methodology, data points, measures, stimulus) between studies in the final article group following full-text screening, and acknowledging the current lack of research in this particular field [3,56], assessment of bias [74] using tools such as the ‘Standard Quality Assessment Criteria for Evaluating Primary Research Papers from a Variety of Fields’ [75] was not considered beneficial by the authors (SJM, AGD) for this review, as identified limitations within studies may render seminal results or insights ineligible. As a substitute to this assessment, we qualitatively addressed study limitations and bias in the discussion section of this manuscript to help provide a framework for future research to improve the current knowledge base. Furthermore, the structuring of our methods will likely serve to help reduce bias in future research.

### 2.5. Data Synthesis

A structured narrative synthesis approach [76] was agreed upon between the researchers (SJM, AGD) for this review as it is sympathetic to the synthesis of findings and results from studies with diverse features (e.g., study design, methodologies, samples, and measures). Familiarization of each included article through full-text screening and the data extraction process enabled the generation of key research themes to assist the reviews analytical narrative thus ensuring the full scope of the data is captured.

## 3. Results

The total studies included in this qualitative assessment following full-text screening adhere to the inclusion and exclusion criteria (See Eligibility Criteria). The majority of ineligible articles did not directly analyze sound and its effects on symptoms of *SI* post-awakening, were qualitatively analyzed, or were unoriginal research (Figure 1). The geographical location of the research included was primarily conducted in English speaking, Western nations. Five were conducted in the United States of America [77,78,79,80,81]; five in Australia [82,83,84,85,86]; one in France [58] with one in Japan [57]. The publication dates of all articles range from 1992 to 2020 with five published between 2019 and 2020, indicating the increased interest in the topic. Eight studies [57,58,77,78,79,80,81,86] were conducted in laboratory settings (Controlled, nonblinded, repeated measures [*n* = 1]; Controlled, non-blinded [*n* = 1]; Randomized, nonblinded, repeated measure designs [*n* = 3]; Randomized, non-blinded [*n* = 1], and non-blinded observational studies [*n* = 2]), and four in the field (Controlled, non-blinded, repeat measures [*n* = 1]; Non-blinded, repeated measures [*n* = 2], Blind questionnaire [*n* = 1]). Participant sample size ranged from *n* = 16 to *n* = 188 in the laboratory studies, and *n* = 14 to *n* = 50 for the field studies. Six studies [77,78,79,80,81,82] tested children participants ranging in age between 5 and 16 years old, one study tested children and adults (6–59) [86], and five studies analyzed adult participants aged from 18 years and above. One study tested males only [58], whilst the remaining eleven studies tested included participants of all gender specifications. Eight articles do not specifically report participant sleep duration [77,78,79,81,82,83,85,86] and eight did not report awakening time [77,78,79,80,81,83,84,85]. Observed differences and combinations of awakening measurement techniques included: EEG-confirmed sleep-stage at awakening (*n* = 8); Non EEG-confirmed awakenings (*n* = 4; Predetermined awakening time [*n* = 2]; Natural awakenings [*n* = 2]). Test stimuli vary in type and context between studies. Eight studies examined sound in an emergency awakening scenario with audio treatments that include domestic fire alarms, human voice, or hybrid combinations [77,78,79,80,81,82,85,86]. Three studies examine music [57,83,84], and one tested noise [58]. Alternate stimulus includes an intercom (*n* = 1) [57] multi-modal conditions which combine sound and vibration (*n* = 1) [80], and voice and alarm (*n* = 1) [79]. Ten of the studies reported on stimulus loudness using the decibel scale (dB; perceived as loudness), where readings for the stimulus treatments ranged between ~ 60 dB and 100 dB (60 dB [*n* = 3]; 75 dB [*n* = 1]; 85 dB [*n* = 3]; 89 dB [*n* = 1]; 100 dB [*n* = 2]). A subjective 5-point self-report of loudness presented in one article ranged from ‘Low volume’ to ‘Very high in volume’ with 44% reporting ‘Neither high-nor low in volume’ [83]. The objective and subjective measures employed in the articles differ markedly. All but one study employed objective measures [83], while six did not record subjective measurements [58,77,78,79,80,81].

To assist this review, three primary themes were identified for synthesis which encapsulate the entirety of the auditory stimulus tested within and between studies. These are: Noise [58], Emergency tone sequences; Voice Alarms and Hybrids [77,78,79,80,81,82,85,86], and Music [57,83,84].

### 3.1. Noise

Through the testing of two conditions (Baseline [no nap, no noise; no nap, noise]; Experimental [nap-no noise, nap-noise]) over two separate nights in a counterbalanced design, Tassi et al. [58] aimed to evaluate the effects of a 5000 Hz pink noise signal (similar to white noise with predominantly lower frequencies) delivered at 75 dB on participant spatial memory performance following awakening from 1-h naps placed at 00:00 h and 03:00 h. With noise presented, no difference was observed between reaction time (RT) performance in the Baseline (no-nap) and Experimental (nap) conditions at 01:00 h. Without the presentation of noise, performance was inferior for up to 15 min in the Experimental (nap) condition compared to the Baseline (no-nap). The observed difference between conditions suggests that pink noise may have improved performance by reducing the expected effects of *SI* following awakening. However, these results did not translate during the later test session, as pink noise was shown to be ineffective and potentially exacerbate performance decrement. These conflicting findings between test epochs and pink noise effects are hypothesized to be a potential result of sleep stages upon awakening; however, at the preliminary stage of this research, the study authors [58] acknowledge no firm conclusion, thus further research is required. 

### 3.2. Emergency Alarms; Tone Sequences, Voice and Hybrids

Several studies [77,78,79,80,81,82,85,86] have analyzed fire alarms and their influence on human performance following abrupt awakenings in pseudo-emergency scenarios. Within these investigations a variety of experiment designs and stimulus comparisons have been made, and a clear focus on the age demographics of participants has been pursued, specifically children/minors (6–17 years) and adults (18+ years).

#### 3.2.1. Children

The first study to analyze the effect a smoke detector alarm exhibits on child/juniors (6–17 years [Mean 11.6 years]) awakenings and subjective *SI* was conducted by Bruck [86] in 1999. In this research, the author simultaneously tested the parents/guardians to enable an age group comparison between results. Conducted in residential conditions over a four-night program with tests occurring on the second and third nights without the participants’ knowledge, the thirty-six subjects (22 children) were exposed to an alarm at 60 dB between 01:00 and 04:30 h. Through the collection of each subject’s objective and subjective data (wrist actigraphy, self-report questionnaires) upon and post-awakening, the results reveal that 85% of the children’s group slept through one or both of the alarm presentations, while 100% of the adults consistently awoke to both. For successful awakenings, 95% of all subjects did so within 32 s of the alarm sounding. Concerning ratings of subjective *SI* (clear-headedness), no significant difference was observed between groups. Across the test groups, an average rating of ‘moderately clear-headed’ was reported immediately following awakening; however, no significant difference was reported between subsequent test points (1. At first alarm hearing; 2. ~3 min after awakening; 3. 4–7 min after awakening). The mean Karolinska Sleepiness Scale (KSS) values for both test nights corresponded to an equivalent ranking of neither sleepy nor alert. This seminal study reveals that a smoke alarm deployed at 60 dB is inferior in awakening younger participants than older; however, for successful awakenings across groups, the subjective measures of *SI* were reported to be not significantly different [86].

In subsequent research, Bruck and Reid [82] compared results between three independent field studies she and her colleagues had previously undertaken which tested a mother’s and female actor’s voice relaying an escape notification (Mother: “There is a fire! Wake up now! Quickly go outside! Actor: “Danger! There is a Fire! Wake up now and go and investigate!” [315 Hz–2500 Hz]) (Study 1); a low pitch Temporal three (T-3) signal (500 Hz) (Study 2), and a high pitch Australian standard ‘pulse’ signal (~4000 Hz) on child participants deployed at ~89 dB (Study 3). In Study 1 participants were alerted at 01:00 h, while Studies 2 and 3 alerted subjects at 01:00 h and 03:00 h. Ratings of clear-headedness were recorded at three different time points (i.e., when the alarm first went off; when the subjects exited their bed; when completing the questionnaire outside the bedroom) and an adapted KSS was completed ~7 min following awakening. Across studies, successful awakenings were statistically significantly inferior for the Australian standard signal compared to the voice and low pitch T-3 signals. Similarly, participant sleep latency was significantly greater on arousal when awakening to the standard alarm. The analysis of subjective *SI* (clear-headedness) revealed no significant difference between alarm type (with a majority of rankings between 2 [quite a bit clear-headed] and 3 [moderately clear-headed]), or improvement over time. Further, the mean KSS sleepiness ratings of all alarms correspond to an approximate rating of 2 (sleepy but no difficulty staying awake) and less than 3 (neither sleepy nor alert); however, no statistically significant difference was observed between treatments. Concerning the effectiveness of emergency alarm tone sequences on objective performance in children, this analysis demonstrates that a high-pitched signal ‘pulse’ appears to be less effective in arousal than a mixed temporal sequence with lower frequencies. Additionally, all reported stimuli in this study show evidence of a positive effect on reducing subjective *SI* (clear-headedness) with a moderate influence on perceived sleepiness. 

Researchers Smith, Splaingard, and colleagues have together produced a collection of studies which build on and refine Bruck et al.’s [82] original research focus. The first study [77] compared the effectiveness of a 100 dB parent voice alarm (“First name! First name! Wake up! Get out of bed! Leave the room!”) with a high pitch T-3 tone alarm (~4000 Hz) in prompting participants to awaken and perform a self-rescue procedure. Conducted in a laboratory setting following a randomized, unblinded design, each subject was awakened during the first two-stage 4 sleep cycles (S4S). The results in this study reveal that the voice treatment significantly outperformed the high pitch T-3 alarm in awakening participants. Additionally, the subjects were significantly more likely to complete the escape procedure within 5-min and 3-min time bins from awakening to the parent voice alarm than the high pitch temporal signal.

Conducted under a similar protocol to Smith et al. [77], the previous year, Splaingard et al. [80] analyzed RT performance following awakening from S4S to a loud (100 dB) parent voice alarm message and T-3 signal, with the addition of a hybrid alarm version (T-3 and vibration wand placed under the pillow). In all conditions participant RTs were significantly slower than baseline readings; however, the hybrid alarm produced significantly faster RTs than parent voice and tone-only alarm. There was no significant difference reported for performance between the parent voice and tone alarm. Furthermore, the RTs of all treatments following the first S4S awakening were significantly shorter that the second awakening. With respect to awakenings and escape procedure performance, no significant different was reported between subjects who successfully awakened and escaped to those who did not.

More recent studies by Smith et al. [78,79,81] continue this line of inquiry by the repeated testing of participant performance when awakened from S4S and completing a self-rescue sequence. The first [78] assessed the effectiveness of three maternal voice alarms: Name only (“Name, Name! Name, Name! Name, Name!”); Instructions only (“Name, Name! Wake up! Get out of bed! Leave the room!”), and Name and Instructions comparative to a high pitched (~3200 Hz) T-3 residential fire alarm. The second [79] compared a female and male voice reciting the escape instructions delivered in the previous study (“Fire! Fire! Wake up! Get out of bed! Leave the room!”), a hybrid alarm consisting of a low-frequency T-3 (500 Hz) signal plus a female voice delivering the instructions, and the high pitch T-3 sequence deployed in the first study. The third [81] examined two female voice (Mothers; Female actors) alarm messages as presented in studies 1 and 2, compared with the low and high-frequency T-3 tonal sequences. All stimuli in each study were presented at 85 dB.

The results across all three studies revealed that a high pitch T-3 alarm was significantly less effective in prompting awakening and a self-rescue procedure compared to the entire spectrum of experimental treatments tested. Within studies, no significant difference was observed between each variant of the maternal voice alarm messages (Name only; Instructions only; Name and Instructions) tested in the first study. Likewise, in the second study, no significant differences where observed between the male voice notification, female voice notification, and a novel hybrid (voice and low-frequency T-3) alarm. Lastly, the third study showed that a low-frequency alarm (T-3; 500 Hz) significantly improved awakening and escaping time compared to a maternal voice alarm. When compared to an actor’s voice alarm the low-frequency alarm (T-3; 500 Hz) significantly improved awakening time, though not escaping. No significant differences were observed between awakening and escaping in the mother’s or stranger’s voice alarm treatments.

In summary of the research reviewed in this sub-section, symptoms of *SI* in children appear to be heightened when awakening to a high-frequency alarm compared to a voice, or low-frequency alarms (500 Hz). Additionally, both the voice (Maternal Male; Actors) and low-frequency T-3 alarm types appear comparable in effectiveness against symptoms of *SI*. However, further research with sufficient group sizes for power to resolve the potential interaction effects between the multiple levels of the various factors is required to establish firm conclusions.

#### 3.2.2. Adults

Through a novel approach incorporating naive (unprepared for the first alarm) and non-naive (prepared for the second alarm) conditions, Bruck and Horasan [85] investigated the awakening effectiveness and post-awakening objective performance of young adult participants exposed to a high frequency ‘pulse’ sequence (2000–4000 Hz) smoke alarm (~60 dB). Subjects were allocated to one of three test groups (Stage 4, Stage 2 or REM sleep) where the alarm signal was sounded twice (naive and non-naïve, respectively) during the test session. Following awakening to the naive signal the non-naive stimulus was triggered as soon as each subject returned to their allocated sleep stage. Upon each awakening, all participants completed subjective measures (including Sleep quality and Sleep quantity) together with a computer reaction time (RT) performance task. The results from this inquiry report no significant differences for all sleep stage awakenings between the time to achieve full wakefulness in both conditions (naive; non-naive). Furthermore, no significant decrement in subject RT performance was observed following awakening compared to the control measures (before sleep and a test following a morning shower). Considering *SI*, the results from this study suggest that a high frequency ‘pulse’ alarm (2000–4000 Hz; ~60 dB) is as successful in awakening participants and reducing symptoms of SI regardless of sleep stage at awakening compared to baseline readings. However, as there are no comparisons to a control condition or different signal designs as tested with children, the most appropriate alarm sound elements (e.g., frequency and/or volume) in context are yet to be determined.

### 3.3. Music

Hayashi et al. [57] and McFarlane et al. [83,84] have both explored the potential for music to counteract *SI*. 

Hayashi et al. [57] investigated the impacts sound preference may have on *SI* following a 20-min daytime nap at 14:00 h. The Experimental group was awakened with a high or low-preference stimulus (60 dB) which persisted until the completion of the test. The Control group was awakened by an intercom. High-preference stimulus was nominated by the participants and consisted of audio described as ‘popular music’. The low-preference stimuli (described as ‘excitative’ music by the authors) was selected by the researchers (‘Mars’ from ‘The Planets’ by Gustav Holst; 60 dB). Upon waking, participant performance was measured by completing a visual oddball task (Experimental group) or a memory search task (Control group) and reporting subjective sleepiness and comfort. The subsequent results show that the high-preference treatment produced improved RT performance compared to the low-preference treatment; however, there was no significant difference considering correct responses as a factor. Subjective sleepiness post-nap was significantly reduced in the high-preference condition when compared to the no-music (intercom) condition and the low-preference stimulus. No significant difference was observed between the low-preference and Control condition. Lastly, subjective comfort was superior in the high-preference condition than either the Control or low-preference stimulus. No significant difference was detected between the low-preference condition and the Control group. Taken together, these results suggest that participant preferred music may counteract symptoms of *SI* (RT; subjective sleepiness), and that subjective comfort may benefit when referenced against intercom type sounds or slower-paced orchestral compositions. However, beyond subjective preference, an understanding for how music may affect *SI* is difficult to extract from this study due to the unspecified musical details of the high-preference and Control stimulus tested, and that no analysis was reported between participant performance following awakening to the intercom.

With a similar research focus to Hayashi et al. [57], McFarlane et al. (2020) reports two studies [83,84] exploring the potential impacts of sounds used for awakening in ecologically relevant, day-to-day scenarios by using remote testing to enable participants to experience treatments in their normal sleeping environment. Through the deployment of an anonymous, self-report online questionnaire, the first study [83] aimed to understand from a ‘bottom-up’ approach how a particular sound or music chosen for awakening may counteract *SI*, and what attributes of these sounds may contribute to the perceived reduction in *SI*. The study’s results did not reveal any significant association between *SI* and the reported waking sound type and the subject’s feeling towards their preferred waking sound. However, the study found that a sound which was ranked as melodic showed a significant relationship to reports of reductions in perceived *SI* as measured by an adapted Sleep Inertia Questionnaire (SIQ) contained in the study. Conversely, sound rated as neutral (neither unmelodic nor melodic) returned a significant relationship to increases in perceived *SI*. The secondary analysis of the study also revealed that a sound rated as melodic was considered to be more rhythmic than a melodically neutral interpretation. Through the analysis of the perceived effects of awakening sounds on *SI*, the findings from this study do indicate that the melodic content of a composition appears to be a potentially important factor for consideration in the understanding and future design of music and its ability to counteract *SI*. 

A second study by McFarlane et al. [84] further interrogated the findings presented in the first study by testing custom-designed and composed melodic and rhythmic stimulus on *SI* for participants awakening in their habitual environments. All stimuli in this study shared musical characteristics (105 BPM; 4/4 m; Key of C; Timbre [woodblock; vibraphone]) to assist with result interpretation. Participants completed an online Psychomotor Vigilance Test (PVT) and questionnaire (including subjective measures KSS and Sleep quality) in two separate test sessions immediately following awakening from nocturnal sleep. Both groups responded to a Control stimulus in the first session, while in the second session, one experienced a Melodic treatment, and the second a Rhythmic treatment. The results show that the melodic treatment significantly decreased attentional lapses, false starts, and had a significantly improved PVT performance score than the control; however, there was no significant difference in RT or response speed (RS) compared to the Control. By contrast, for the Rhythmic test group, there was no significant difference observed for the PVT metrics. The results from this analysis supported and extended the initial results observed in the authors’ previous study [83] and for melodies’ potential to counteract symptoms of *SI* following waking from nocturnal sleep in ecological conditions.

## 4. Discussion

In the current manuscript, the authors employed the PRISMA systematic review guidelines to identify peer reviewed research published in English on how auditory stimulus may influence participant *SI* upon and post-awakening. This process resulted in twelve studies being identified as meeting the criteria (Figure 1), and detailed reading of these studies provided further information on the types of stimuli investigated and their potential efficacy. The following summary thus serves to help highlight areas for future research to improve the current knowledge base of how different auditory treatments may influence *SI*.

### 4.1. Summary of Evidence

The results of this systematic review are derived from the analysis of twelve studies retrieved from three scientific databases (ProQuest, PubMed, Scopus) and through an author publication search (Google Scholar) and subsequent screening (Figure 1). Three primary themes encapsulate the diversity of stimulus reported in the results, including (i) Noise [58], (ii) Emergency alarms; Tone Sequences, Voice and Hybrids [77,78,79,80,81,82], and (iii) Music [57,83,84] (Table 1). The context in which participants have been assessed may be further categorized into two themes, namely, emergency [58,77,78,79,80,81,82,85,86], and non-emergency awakenings [57,83,84]. In both categories, a variety of testing methodologies have been applied within laboratory [57,58,77,78,79,80,81,85] and field [82,83,84,86] settings. Across studies, the participant sample size ranged from *n* = 14 to *n* = 188 (Table 1). The majority of articles investigating emergency awakenings have tested child participants [77,78,79,80,81,82], while in non-emergency awakenings only adults have been assessed [57,83,84]. 

From the results reported on emergency responses in children, the evidence suggests that a low-frequency T-3 signal and voice notifications, regardless of priming or gender [78,81], are more effective in awakening and post-awakening performance than high-pitched alarms [77,78,79,81,82]. When comparing low-frequency and voice notifications, a low-frequency alarm has been shown to be significantly more effective than a maternal voice alarm in post-awakening performance, and significantly more effective than an actor’s voice alarm arousal; however there is insufficient evidence at present to draw firm conclusions between their effectiveness with respect to *SI* [81]. Therefore, research to date indicates that an alarm design employing a T-3 (500 Hz square wave frequency) alarm and voice notifications are superior in counteracting the effects of *SI* in children following abrupt awakenings than alarms with higher frequencies (e.g., ~2000–4000 Hz; Pulsed or Sequenced).

Initial results have been reported concerning auditory emergency awakenings and post-awakening performance in adults’, though due to the observed absence of research that has been undertaken in this domain, the results must be interpreted with restraint. A high-frequency ‘pulse’ alarm (2000–4000 Hz; ~60 dB) has been shown to be as successful in awakening participants and reducing symptoms of *SI* regardless of sleep stage at awakening, compared to pre-sleep baseline readings [85]. However, as there are no comparisons to a control condition or different signal designs as tested with children, the most appropriate alarm in context is yet to be resolved. Similarly, Pink noise (5000 Hz; 75 dB) has shown the potential to improve performance following awakening from an early night sleep epoch, though not in later awakenings [58]. Currently there is insufficient evidence to support firm conclusions for Pink noise and its effectiveness in children or adult awakenings as a countermeasure to *SI*. Between children and adult awakenings, there was reported to be no significant difference between demographics in post-awakening RT performance when responding to a high-frequency alarm [86], though due to the limited data available and low statistical power within studies, further research is required to confirm this finding.

Music does show positive results as a countermeasure for *SI* in non-emergency adult awakenings [57,83,84]. The perceived melodicity of a participant’s chosen waking alarm shows a significant relationship to reductions in subjectively measured *SI* [83]. Preference for popular music and stimuli with melodic features have been shown to counteract *SI* in RT and sustained attention [57,84], and that neutral and rhythmic treatments are less effective compared to melodicity [84]. 

### 4.2. Limitations and Recommendations

This review provides the basis for a transparent appraisal of sound, voice, and/or music to counteract *SI* in emergency and non-emergency awakening scenarios considering different age demographics. It is observed from the data extracted that there are several limitations to overcome in order to improve future research in this field. Limitations of this systematic review include the shortage of focused research specific to *SI* post-awakening to audio, and the heterogeneity of the assessed articles regarding study context and design, sample size, stimulus type and reporting, and *SI* assessment and reporting methods (objective and subjective) (Table 1). 

There is an observed bias of the stimuli investigated, however, this is largely a byproduct of the research context (i.e., emergency and non-emergency) which may inform which particular alarm types (Alarm signals, Voice warnings; Music) are to be evaluated. For example, musical approaches have not been assessed within emergency settings, and conversely, alarm and voice treatments are yet to be evaluated in non-emergency settings. Future research may consider a counterbalanced design of alarm types in different contexts to assess the potential effects in contrasting settings. Furthermore, the analysis of auditory countermeasures for *SI* between demographics requires more research, particularly in emergency awakenings. In this context, a bias potentially exists between child awakenings and adults. Compared to what is known for auditory emergency awakenings with different stimuli in children, a knowledge gap exists in adult demographics when assessing audio treatments.

Within laboratory settings, the methodological quality of articles reviewed generally report prudent designs [58,77,78,79,80,81,85]; however, due to the variability of stimuli assessed, the capacity to draw conclusions across studies remains limited. Additionally, investigations conducted in laboratory settings often fail to replicate the ecological conditions in which people typically awaken [87], thus presenting challenges in the interpretation and verification of results with respect to ‘real world’ situations. Field studies provide superior representations of sleep-wake behavior in every-day conditions, but in doing so, there are clear technical limitations observed, specifically with respect to monitoring sleep stage at awakening [82,83,84,86]. For studies investigating *SI* in ecological settings, technological interventions (such as applying EEG sensors) often contradict study objectives, thus, compromises have been made in the data gathering process (e.g., wrist actigraphy or self-report measures) to ensure the ecological validity of such investigations. Considering this challenge, advancements in remote data-gathering technology [88] present opportunities for the refinement of such testing methods by bridging the advantages of both laboratory and field-based studies.

The refinement of the methods for assessing *SI* across the studies reviewed would be advantageous. In both emergency and non-emergency conditions, and in laboratory and field settings, the measuring of *SI* varies substantially in methodology and cognitive metrics. Test batteries which include increased testing points over time post-awakening, and the inclusion of validated objective (e.g., PVT) and subjective measures (KSS, SIQ) would further refine our knowledge for *SI* identification and duration.

Improvements in emergency and non-emergency contexts conducted in laboratory or field conditions would benefit by including controls in the study design to allow future comparisons between studies, stimulus, and research groups. This may include no-noise, specified standardized signals, or experimental stimuli that can be easily replicated. For studies investigating voice warnings future research would benefit through the reporting signal specifications (e.g., phrasing [rhythm, timing], and pitch ranges [Hz]), as this would assist in the comparison between targeted tone alarms, and afford technical discrimination between musical approaches. Indeed, improvements in online open access data reporting are likely to result in better data use and replication [89,90]. Similarly, general reporting of music stimuli such as genre requires greater detail as the ability to compare and validate results can present challenges. Thus, all stimuli tested (including hybrid designs) moving forward should be reported in a manner that affords uncomplicated and accurate replication by other researches in any geographic location.

Lastly, given that the primary objective for this systematic review is to report on auditory countermeasures for *SI* post-awakening, and that the reporting of such results are often uncategorized or secondary objectives, it is conceivable that bias in database search terms and article identification may exist. However, considering the selection criteria of this systematic review, and the contributions made by several article authors to this field, it is likely that the studies presented here do represent the current state of knowledge in auditory countermeasures for *SI* research.

## 5. Conclusions

This systematic review provides an up to date summary of existing original empirical research published in English on auditory countermeasures for *SI* post-awakening. From research spanning 1992 to 2020, the findings illustrate that there are two lines of inquiry which interrogate *SI* including emergency, and non-emergency test scenarios. The results indicate that for children awakening in emergency conditions, a low pitch alarm or voice notifications appear to be more effective in counteracting the effects of *SI*, than alarms with higher frequencies, particularly in memory and reaction time. However, further research is required to confirm an effective difference between low-frequency and voice alarms. Similarly, for adults abruptly awakened, there is currently insufficient evidence to support firm conclusions regarding alarm types and voice signals concerning *SI* post-awakening. Positive results have been found in non-emergency awakenings with respect to musical treatments in adults, particularly preferred popular music, and alarms with melodic qualities. In both categories investigating *SI*, the results observed reflect the potential for sound and music to counteract *SI*. Furthermore, there are identified opportunities to capitalize upon, that in turn will strengthen the knowledge base in this field. These include increasing research efforts in adult emergency awakenings with different stimulus treatments, comparing treatments between demographics, and further exploring musical treatments in children/adult and emergency conditions. Additionally, refining testing methods in field studies will assist in knowledge production for how sound may counteract *SI* in ecological conditions, and help design experiments that might seek to better understand the neurophysiology of how sound is processed by the brain [91] and a mechanistic account of how to reduce *SI*. Acknowledging the potentially detrimental effects resulting from *SI*, the results from this systematic review may be an important reference for researchers and professionals in the fields of auditory perception, sleep and cognitive psychology, and sound design. Industry would also benefit from this systematic review, particularly in first response, transport, and high-risk occupations.

## Figures and Tables

**Table 1:** Chronological summary of studies analyzing auditory alarms and symptoms of *SI* post-awakening.

Author	Setting	*n*	Age	Sex or Gender	Study Design	Sleep Duration	Awakening Time	EEG Confirmed Awakening	Stimulus	dB	Objective Measure	Subjective Measure
Tassi, Nicolas et al. (1992)	Lab	44	19–27	Male	Controlled, nonblinded, repeated measures	1 h	01:00 04:00	Yes	**Control/Baseline:** No-noise **Experimental:** Pink noise	75	**All test groups:** Spatial Memory Test	None
Bruck and Horasan (1995)	Lab	24	18–24	Male Female	Non-blinded, observational study	NR	NR	Yes	**Control/Baseline:** None **Experimental:** High frequency ‘pulse’ sequence alarm	55–65	**All participants:** Reaction time (RT)	**All participants:** Sleep quality Sleep quantity
Bruck (1999)	Field	36	6–59	Male Female	Non-blinded, repeated measures	NR	01:00–04:30	No	**Control/Baseline:** None **Experimental:** Smoke alarm	60	**All test groups:** Wrist actigraphy	**All test groups:** Clear-headedness scale KSS
Hayashi, Uchida et al. (2004)	Lab	16	20–23	Male Female	Controlled, non-blinded	20 min	14:20	Yes	**Control/Baseline:** Intercom **Experimental:** (1) Participant choice (2) Selected by the experimenter	60	**Control group:** Memory search task **Experimental group:** Visual oddball task	**All test groups:** Sleepiness rating Comfort rating
Bruck, Reid et al. (2004)	Field	(S1) 20 (S2) 14 (S3) 14	6–10	Male Female	Non-blinded, repeated measures	NR	01:00 03:00	No	**Control/Baseline:** None **Experimental:** (1) Mothers voice (2) Female actors voice (3) High frequency ‘pulse’ sequence alarm (4) Low-frequency T-3 tone alarm	89	**All test groups:** Wrist actigraphy	**All test groups:** Clear-headedness scale Adapted KSS
Smith, Splaingard et al. (2006)	Clinical	24	6–12	Male Female	Randomized, non-blinded	NR	Awakened during the first two S4S cycles	Yes	**Control/Baseline:** None **Experimental:** (1) Mothers voice (2) High-frequency T-3 tone alarm	100	**All test groups:** Self-rescue sequence	None
Splaingard, Hayes et al. (2007)	Clinical	44	6–12	Male Female	Non-blinded, observational study	Cycle 1: ~65 min Cycle 2: ~50 min Mean time between alarms 76 min	Awakened during the first two S4S cycles	Yes	**Control/Baseline:** None **Experimental:** (1) T-3 tone alarm (2) Mother’s voice (3) Hybrid alarm (T-3 tone alarm and vibration wand)	100	**All test groups:** PVT (10-min) Self-rescue sequence	None
Smith, Chounthirath et al. (2019)	Clinical	176	5–12	Male Female	Randomized, non-blinded, repeated measures	NR	Awakened during the first two S4S cycles	Yes	**Control/Baseline:** High-frequency T-3 tone alarm **Experimental:** (1) Maternal voice (Name only) (2) Maternal voice (Instructions only) (3) Maternal voice (Name and instructions)	85	**All test groups:** Self-rescue sequence	None
McFarlane, Garcia et al. (2020)	Field	50	18+	Male Female	Blind, questionnaire	NR	Following nocturnal sleep	No	Participant specified	Self-report subjective scale	None	**All participants:** Self-report questionnaire including: Sleep Inertia Questionnaire (SIQ) Music element ratings Sound type Feeling toward the sound rating
Smith, Chounthirath et al. (2020)	Clinical	188	5–12	Male Female	Randomized, non-blinded, repeated measures	NR	Awakened during the first two S4S cycles	Yes	**Control/Baseline:** None **Experimental:** (1) Female voice (2) Male voice (3) Hybrid voice-tone alarm (Low-frequency T-3 tone alarm and female voice) (4) High-frequency T-3 tone alarm	85	**All test groups:** Simulated escape procedure	None
Smith, Chounthirath et al. (2020)	Clinical	176	5–12	Male Female	Randomized, non-blinded, repeated measures	NR	Awakened during the first two S4S cycles	Yes	**Control/Baseline:** None **Experimental:** (1) Child’s mother voice (2) Female strangers voice (3) Low-frequency T-3 tone alarm (4) High-frequency T-3 tone alarm	85	**All test groups:** Simulated escape sequence	None
McFarlane, Garcia et al. (2020)	Field	20	18–49	Male Female	Controlled, non-blinded, repeated measures	**Group A & B:** 5–9+	Following nocturnal sleep	No	**Control:** Tonal pulse **Experimental:** (1) Melodic (2) Rhythmic	None	**All test groups:** PVT (3 min)	**All test groups:** KSS Hours Slept Sleep Quality Scale

Table Abbreviations: NR (Not reported); KSS (Karolinska Sleepiness Scale); PVT (Psychomotor Vigilance Test); S4S (Sleep Stage 4); T-3 (Temporal three smoke alarm); dB (Decibel); S1, S2, S3 (Study No).

## Supplementary Materials

Supplementary materials can be found at https://www.mdpi.com/2624-5175/2/4/31/s1. Table S1: PRISMA 2009 Checklist.
